# Identifying Herbal Candidates and Active Compounds for Psoriasis Through Multiscale Network Analysis

**DOI:** 10.3390/cimb46110712

**Published:** 2024-10-25

**Authors:** Gi-Beom Kim, Su-Yeon Lee, Soon-Woo Shin, Il-Joo Jo, Ji-Hwan Kim, Seungho Lee, Won-Yung Lee

**Affiliations:** 1 College of Korean Medicine, Wonkwang University, Iksan 54538, Republic of Korea; 2 Research Center of Traditional Korean Medicine, Wonkwang University, Iksan 54538, Republic of Korea; 3 Department of Sasang Constitutional Medicine, Division of Clinical Medicine, School of Korean Medicine, Pusan National University, Busan 46241, Republic of Korea; 4 College of Korean Medicine, Woosuk University, Jeon-Ju 54987, Republic of Korea

**Keywords:** psoriasis, multiscale network analysis, herbal candidates, active compounds

## Abstract

Psoriasis is a chronic inflammatory skin disorder characterized by the hyperproliferation of keratinocytes and immune system dysregulation, with significant needs due to the limitations and adverse effects of current treatments. In this study, we sought to discover novel herbal candidates and their active compounds for psoriasis by leveraging a multiscale network analysis. We conducted a comprehensive analysis of data from 348 medicinal herbs and their active compounds, identifying *Piperis longi fructus*, *Pini koraiensis semen*, *Schisandrae fructus*, and *Cnidi fructus* as top candidates without reported evidence. Key active compounds, such as piperine, piperlongumine, α-humulene, schizandrin A, schizandrin II, and torilin, were prioritized for their ability to target psoriasis-associated proteins, including STAT3, TNF, IL-6, and NF-κB. These compounds are involved in the modulation of critical inflammatory pathways, notably the MAPK signaling cascade, which plays a central role in psoriasis pathogenesis. Our findings suggest that these herbal compounds may not only mitigate inflammation but also regulate keratinocyte hyperproliferation, addressing fundamental mechanisms underlying the disease. This approach highlights the utility of multiscale network analysis in identifying promising natural therapies, offering new insights and potential avenues for safer and more effective psoriasis management.

## 1. Introduction

Psoriasis is a chronic inflammatory skin disorder characterized by symptoms such as epidermal hyperplasia, silvery-white scales, erythema, and the formation of papules and plaques [1]. Recently, it has been increasingly recognized as a systemic disease due to its association with a higher incidence of comorbidities such as psoriatic arthritis, obesity, diabetes, hyperlipidemia, and hypertension [2]. The prevalence of metabolic syndrome among psoriasis patients is estimated to be between 20% and 50%, with meta-analyses indicating that the risk of metabolic syndrome is at least twice as high in individuals with psoriasis compared with those without the condition [3]. While conventional treatments such as methotrexate, acitretin, and cyclosporine are widely used for managing psoriasis, their therapeutic efficacy is often limited, with Psoriasis Area and Severity Index 75% (PASI 75) response rates typically ranging from 50% to 70%, leaving many patients without satisfactory outcomes. Furthermore, these treatments are associated with serious side effects, including hepatotoxicity and hematologic abnormalities with methotrexate, hepatotoxicity and teratogenicity with acitretin, and nephrotoxicity with cyclosporine. These risks necessitate the careful selection of treatments tailored to individual patient conditions [4]. Although biologics have recently emerged as alternatives, challenges remain regarding the sustained efficacy and potential risk of adverse reactions. Given these challenges, there is an urgent need for research into the development of effective and safe therapeutic options for psoriasis. The long-term use of existing treatments poses significant challenges in patient management due to their severe side effects, underscoring the demand for safer and more sustainable alternative therapies.

Herbal medicine, particularly within the framework of traditional Korean medicine, has demonstrated potential as a safe and long-term treatment option that may effectively alleviate symptoms while minimizing the side effects associated with conventional therapies [5]. Clinical studies, such as those by Xing et al., have suggested that herbal medicine may exhibit anti-inflammatory and immunomodulatory effects in the treatment of psoriasis [6]. However, these studies often present incomplete clinical findings related to the effects of complex prescriptions. While these results indicate potential mechanisms of herbal medicine in improving lipid metabolism, reducing inflammation, and modulating the immune system in psoriasis treatment, there is still a lack of systematic research methodologies to thoroughly explore and validate these mechanisms [7]. Employing more systematic research methodologies is imperative for optimizing the therapeutic potential of herbal medicine in the management of psoriasis.

Network pharmacology is an approach based on bioinformatics and systems biology that investigates the mechanisms and interactions of drugs through the network of diseases, gene targets, and compounds [8]. This approach addresses the limitations of traditional single-target strategies by considering multi-target interactions within the network as part of drug development strategies [9,10]. Network pharmacology thus represents a suitable method for exploring the efficacy of natural products and herbal medicines, which are characterized by multi-compound and multi-target properties, and providing deeper insights into their mechanisms of action [11]. For instance, through network analysis, molecular docking, and experimental validation, the mechanisms of *Curcuma* in treating ulcerative colitis have been elucidated [12]. Additionally, a comprehensive network analysis has contributed to the understanding of the pharmacological mechanisms of the herb pair Huanglian Ganjiang in colorectal cancer [13]. These examples demonstrate the potential of network pharmacology in analyzing the complex interactions between drugs and diseases, making it an effective approach for exploring the active compounds and mechanisms of herbal medicines.

**Figure 1:**
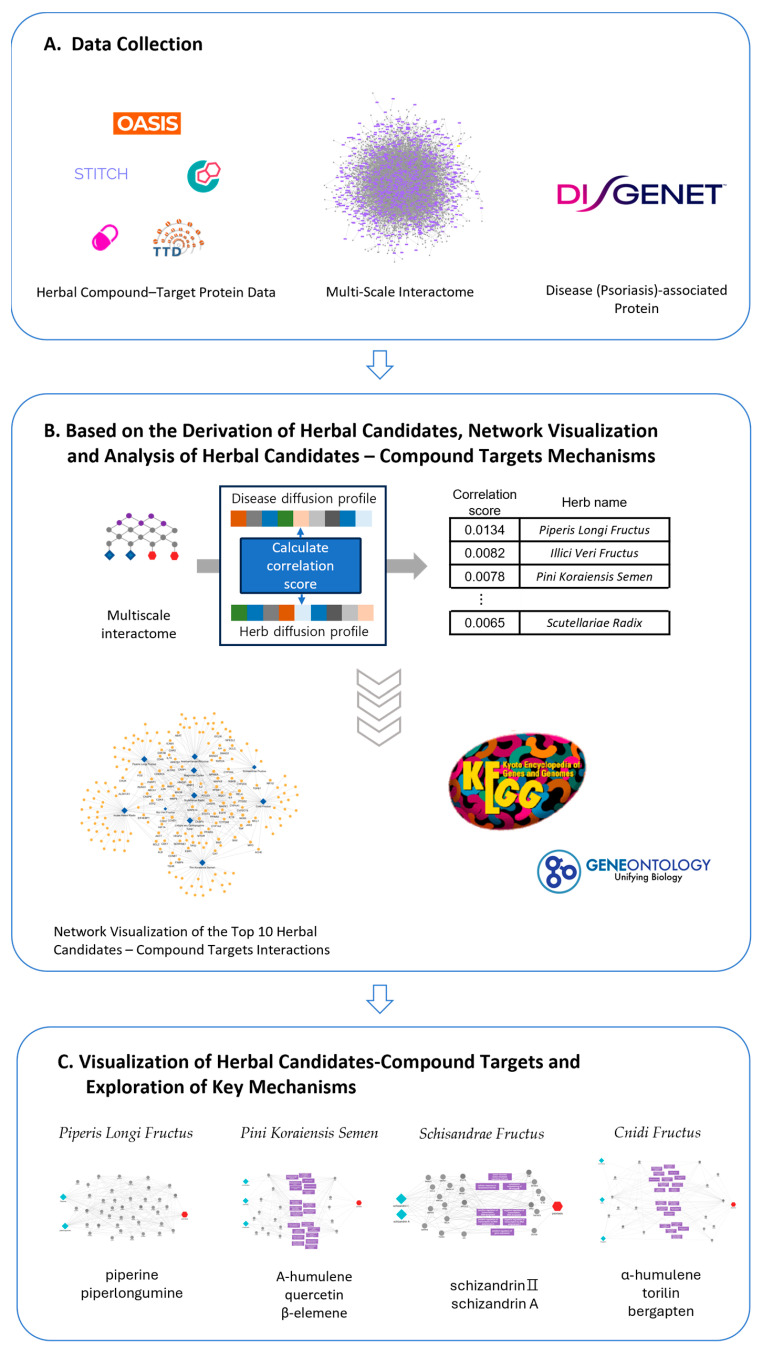
Integrated framework for investigating herbal candidates, their active compounds, and mechanisms in psoriasis treatment. (**A**) Data collection. Herbal compound–Target proteins, multi-layer interactome, and psoriasis-associated proteins were collected from multiple datasets and databases. (**B**) Correlation score-based prioritization of herbs related to psoriasis treatment, followed by visualization and mechanism analysis. Correlation scores were calculated to screen potential herbs for psoriasis treatment under the multiscale interactome. The top 10 herbs with the highest correlation scores were selected and visualized. Then, Gene Ontology (GO) analysis and Kyoto Encyclopedia of Genes and Genomes (KEGG) pathway analysis were conducted to investigate the biological functions and pathways. (**C**) Herbal candidate–Compound targets visualization and mechanism exploration. These processes were conducted using Cytoscape to investigate the influence of herbal candidate–compound targets on psoriasis treatment mechanisms.

Based on the potential of network pharmacology, this study aims to identify new herbal candidates beneficial for psoriasis through a multiscale network-based framework (Figure 1). The advantage of a multiscale network analysis is its ability to consider the propagation effects across various networks, including those related to biological functions [14]. This allows for more precise predictions of therapeutic effects and key mechanisms. To achieve this, 348 herbs, their compounds, protein targets, and psoriasis-related protein targets were collected from various databases. These datasets were integrated to construct a multi-layer network, allowing us to observe the influence of specific herbs and their compounds within the network. The simulated impact, defined as the diffusion profile of compounds and diseases, was used to prioritize herbal candidates believed to be effective in treating psoriasis. We carefully selected herbs based on a review of previous studies on their efficacy and further investigated the active compounds of high-priority herbs from reported studies. Finally, the mechanisms of unreported compounds, which ranked highly within the multiscale network, were analyzed. Through this approach, we aim to identify new herbs and compounds effective for psoriasis, highlighting the potential of multiscale network analysis and providing a foundation for future research into the mechanisms of herbal compounds.

## 2. Materials and Methods

### 2.1. Construction of the Herbal Compound–Target Network

#### 2.1.1. Herbal Compound Dataset Construction

In this study, the OASIS Korean Medicine Database (https://oasis.kiom.re.kr/index.jsp, accessed on 15 April 2024), operated by the Korea Institute of Oriental Medicine (KIOM), was employed to collect compound information from herbal medicines [15]. The physicochemical compound data were retrieved from the “Herbal Medicine-Physicochemical” section of the OASIS platform, yielding a total of 16,117 chemical compounds from 478 herbal medicines. To ensure meaningful compound interactions, only herbs containing more than five compounds were considered for further analysis.

#### 2.1.2. Construction of the Compound–Target Dataset

Protein target data were obtained in a manner similar to previous studies [16]. Specifically, the identification of protein targets was based on datasets containing experimentally validated compound–target interactions. These datasets were compiled from credible sources such as DrugBank [17], the Therapeutic Target Database (TTD) [18], the Search Tool for Interactions of Chemicals (STITCH) [19], and the study by Huang et al. [20]. The DrugBank 5.0 database was referenced for drug–target information, providing comprehensive data on established and potential targets, associated diseases, biological pathways, and drugs targeting these proteins. Additionally, STITCH aggregated target data for over 430,000 chemicals across multiple sources. The study by Huang et al. contributed data on both direct and indirect interactions between natural product-related compounds and proteins from an extensive database.

#### 2.1.3. Integration of Herbal Compound and Compound–Target Data, and Network Construction

The herbal compound data from the OASIS database were used as the foundation, and compounds with specified PubChem CIDs were selected for comparison with the compound–protein target data. These datasets were then integrated. Herbal medicines containing fewer than five compounds were excluded, and the final network was constructed to represent the relationships between herbal medicines, their compounds (PubChem CIDs), and protein targets. Then, the analysis considered cases where specific protein targets were affected by both individual and multiple compounds. The top 50 targets for each herbal medicine were selected by calculating the number of pathways linked to each compound. The resulting network was designed to efficiently visualize and analyze the interactions between herbal medicines, chemical compounds, and protein targets.

### 2.2. Enrichment Analysis

A gene set enrichment analysis (GSEA) was conducted using Enrichr (http://amp.pharm.mssm.edu/Enrichr/, accessed on 8 July 2024) to investigate the signaling pathways or biological functions associated with the herbal targets (or components) [21]. Enrichr utilizes a range of gene set libraries (e.g., Gene Ontology and Kyoto Encyclopedia of Genes and Genomes (KEGG)) to perform an enrichment analysis based on the input gene list, yielding results such as adjusted *p*-values, z-scores, and combined scores. Of these, the combined score is calculated by taking the logarithm of the product of the *p*-value and z-score, thereby integrating both metrics to mitigate their individual limitations and produce more reliable results.

### 2.3. Disease–Gene Interaction Analysis and Protein Network Construction

The DisGeNet database was used to analyze disease–gene interactions, with only high-confidence disease–gene association data selected for this study [22]. These associations were curated by experts from various sources, including UniProt, Comparative Toxicogenomics Database, Orphanet, Clinical Genome Resource (ClinGen), Genomics England PanelApp, Cancer Genome Interpreter (CGI), and the Psychiatric Disorders Gene Association Network (PsyGeNET). To maintain the accuracy of the analysis, data inferred from animal models or automatically mined from the literature were excluded. Disease–Gene interactions that were indicated as therapeutic were also omitted from the analysis. Furthermore, only the protein products of genes that will be part of the multiscale interactome network, discussed in the following sections, were selected. This filtered dataset was then used to identify and analyze psoriasis-related proteins.

### 2.4. Multiscale Interactome

The multiscale interactome utilized in our study was based on data constructed by Ruiz et al. [14]. This network was developed by integrating three types of interactions: protein–protein interactions, protein–biological function interactions, and biological function–function interactions. For protein–protein interactions, the network included 387,626 physical interactions among 17,669 proteins, with data collected from major databases such as the Biological General Repository for Interaction Datasets, the Database of Interacting Proteins, and the Human Reference Protein Interactome Mapping Project. Regarding protein–biological function connections, 34,777 associations were established between 7993 proteins and 6387 biological functions, based on data from the human edition of the Gene Ontology database. Finally, the interactions between biological functions were structured into a hierarchical framework, comprising 22,545 associations among 9798 distinct biological functions.

### 2.5. Protein Overlap

A hypergeometric test was performed to evaluate whether the overlap between the protein targets of herbal components and the disease targets of psoriasis was statistically significant or a result of random chance. The *p*-value was calculated to determine if the observed overlap between these herbal components’ protein targets and psoriasis-related proteins exceeded what would be expected by random chance.

P(k, M, n, N)=1−∑i=0k−1niM−nN−iMN

The *p*-value was calculated based on the number of psoriasis-related proteins among the herbal component targets (*k*), the total number of herbal component targets considered in the study (*n*), the total number of psoriasis-related proteins (*N*), and the number of herbal component targets (*M*).

### 2.6. Diffusion Profile Calculation and Analysis

Disease-related active components were identified by calculating and analyzing diffusion profiles, which represent the effects of components or diseases on proteins and biological functions. Previous studies have demonstrated that network-based approaches are effective in uncovering key mechanisms and therapeutic effects of active compounds in various herbal medicines. For instance, such analyses have revealed core mechanisms underlying the therapeutic effects of *Panax ginseng* and polyphenols as well as the antioxidant properties of *Bupleuri radix* [11,23,24]. Diffusion profiles were derived using a matrix-based approach combined with power iteration.

$$r^{\left(k+1\right)}=\left(1-\alpha \right)s+\alpha (r^{\left(k\right)}M+s\sum_{j\in J}r_{j}^{\left(k\right)})$$

The diffusion profile at the k-th state was represented by $r^{\left(k\right)}$, with α denoting the probability that a walker continues to move, and s represented the restart vector, indicating the probability of a walker moving to each node upon restarting. M was derived as the biased transition matrix from the multiscale network. The iteration process was continued until the following conditions were met:

$$||r^{\left(k+1\right)}-r^{\left(k\right)}|{|}_{1}>\varepsilon$$

The tolerance parameter, *ε*, was set according to previous studies. The correlation between the diffusion profiles of the drug and the disease was calculated using the following formula:

$$\frac{\left(r^{\left(c\right)}-{\bar{r}}^{\left(c\right)}\right)\cdot \left(r^{\left(d\right)}-{\bar{r}}^{\left(d\right)}\right)}{||{(r}^{\left(c\right)}-{\bar{r}}^{\left(c\right)})|{|}_{2}||(r^{\left(d\right)}-{\bar{r}}^{\left(d\right)})|{|}_{2}}$$

$r^{\left(c\right)}$ and $r^{\left(d\right)}$ were used to represent the diffusion profiles of the component and the disease, respectively. The top k proteins or biological functions most significantly impacted by the component or disease were selected for further analysis. A network was constructed using the selected entities to emphasize their importance, while component targets not linked to disease-associated proteins or biological functions were excluded. Entities that ranked highest in the diffusion profiles were considered to exert the most significant impact and were therefore deemed critical for therapeutic intervention. The value of k was set to 20 to capture a substantial proportion of the visit frequency within the diffusion profiles. Moreover, the parameter settings for the multiscale interactome were selected based on previous studies, which utilized values such as $w_{drug}=3.21$, $w_{disease}=3.54$, $w_{protein}=4.40$, $w_{higher-level\;biological\;function}=2.10$, $w_{lower-level\;biological\;function}=4.49$, and $w_{biological\;function}=6.58$ with α=0.860, following the same principles established in previous works.

## 3. Results

### 3.1. Exploration of Potential Herbal Candidates for Psoriasis

We constructed a network based on the compound–target associations of 348 herbs and psoriasis-related proteins to identify potential herbal candidates and active compounds for psoriasis treatment. Specifically, a multiscale network analysis was conducted, prioritizing herbs by evaluating protein overlaps and correlation scores with psoriasis-related proteins. The impact (diffusion profile) of each herb on psoriasis was calculated, and the correlation scores were assessed. A hypergeometric test was also performed to evaluate the extent of protein overlap. As a result, herbs showing high correlation scores and significant protein overlaps were identified (Table 1). An enrichment value of five or more between the prioritized herbs and psoriasis-related protein targets was confirmed, demonstrating that the multiscale network-based prediction model effectively identified targets significantly associated with psoriasis. Among the prioritized herbs, *Piperis longi fructus* had the highest correlation score (0.013), followed by *Illici veri fructus* (0.008), *Pini koraiensis semen* (0.0078), *Magnoliae cortex* (0.0077), *Schisandrae fructus* (0.0076), and *Cnidi fructus* (0.0075).

To assess the reliability of the predictions, we investigated existing evidence for the top prioritized candidates. we found that three of the top 10 herbs—*Illici veri fructus*, *Magnoliae cortex*, and *Scutellariae baicalensis*—have previously been reported to exhibit therapeutic effects against psoriasis. Specifically, *Illici veri fructus* has been shown to regulate the IFN-γ signaling pathway, indicating its potential in the treatment of IFN-γ-dependent inflammatory skin conditions such as psoriasis [25]. *Magnoliae cortex* was demonstrated to suppress pro-inflammatory cytokine expression in psoriasis-like animal models [26], while *Scutellaria baicalensis* (also known as *Scutellariae radix*) was found to alleviate psoriasis-like lesions by modulating interactions between keratinocytes and macrophages [27]. The identification of *Illici veri fructus*, *Magnoliae cortex*, and *Scutellaria baicalensis* among the top candidates, all of which have known therapeutic effects against psoriasis, further validates the reliability of the predictive model employed in this research. Meanwhile, herbs such as *Piperis longi fructus*, *Pini koraiensis semen*, *Schisandrae fructus*, and *Cnidi fructus*, which have not been previously associated with psoriasis treatment, displayed high correlation scores and significant protein overlaps (*p*-value < 0.05). These results suggest that these herbs could be novel candidates for psoriasis treatment.

### 3.2. Network Visualization and GSEA of Herbal Candidates for Psoriasis

**Figure 2:**
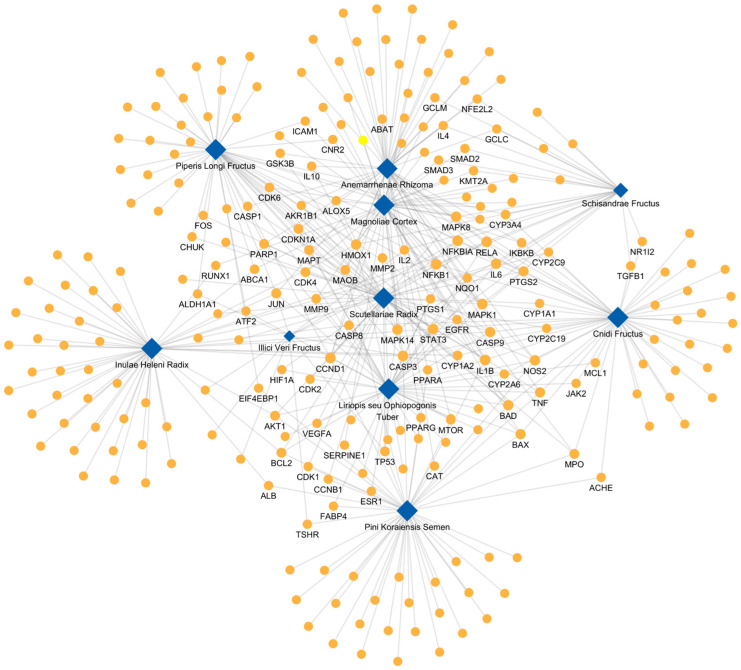
Herb–Target protein network of the top 10 identified herbal candidates for psoriasis treatment. This network consists of 434 interactions between 10 herbal candidates and 254 target proteins. Among them, 84 proteins were found to be shared by at least two herbs. Blue diamonds represent herbal candidates, and yellow circles represent target proteins.

To investigate the mechanisms of the top 10 herbs that exhibited high correlation scores with psoriasis diffusion profiles, a visualized herb–target network was constructed (Figure 2). This network comprised 434 interactions between 10 herbs and 254 targets. Among these, 84 proteins were targeted by at least two herbs, suggesting that these proteins are closely linked to the core mechanisms of the herbs.

Additionally, using the KEGG signaling pathway database, an enrichment analysis of the core targets of the herbs was conducted, focusing on pathways involved in the pathological mechanisms of psoriasis. The results were ranked by the odds ratio (Table 2). The analysis revealed that the TNF signaling pathway, known for inducing inflammatory responses, had the highest odds ratio of 30, with 20 core targets of the herbs being associated with this pathway. Other key signaling pathways identified included osteoclast differentiation, involved in bone resorption; NF-κB signaling, which regulates inflammation and immune responses; the cell cycle, associated with the proliferation and differentiation of skin cells; and PPAR signaling, which plays a role in regulating skin cell proliferation and inflammation. These findings suggest that the herbs with high correlation scores are involved in multiple signaling pathways related to psoriasis, indicating their potential relevance in the context of the disease.

### 3.3. Gene Ontology Enrichment Analysis of Key Protein Targets

**Figure 3:**
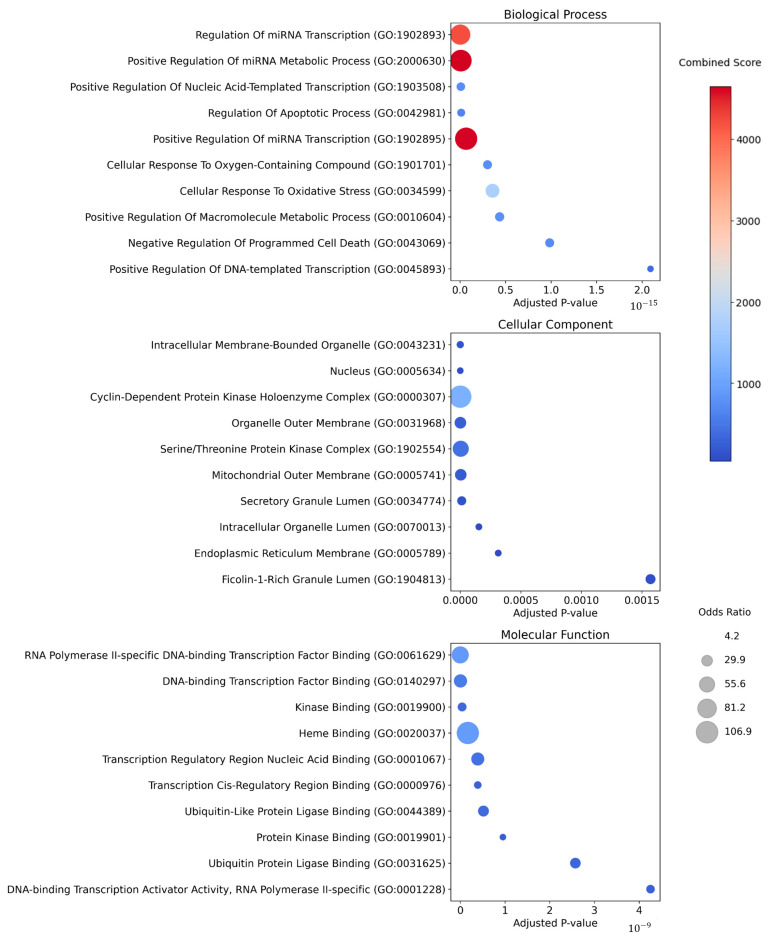
Gene Ontology enrichment analysis of target proteins from prioritized herbal candidates for psoriasis. This figure shows the GO enrichment analysis of target proteins related to prioritized herbs for psoriasis treatment, categorized into Biological Process, Cellular Component, and Molecular Function. Each bubble represents a GO term, with its size indicating the odds ratio (enrichment strength) and its color reflecting the combined score (based on the *p*-value and odds ratio). Combined score: darker red indicates a higher enrichment of the GO term with the target proteins. Odds ratio: larger bubbles suggest stronger associations between the GO term and the targets.

We performed a Gene Ontology (GO) enrichment analysis on the proteins commonly targeted by at least two of the ten prioritized herbs, utilizing the GO Biological Process, Molecular Function, and Cellular Component databases. The top 10 GO terms based on “Adjusted *p*-value” were visualized for each category (Figure 3). Within the Biological Process category, terms such as regulation of miRNA transcription (GO:1902893), positive regulation of miRNA metabolic process (GO:2000630), and positive regulation of miRNA transcription (GO:1902895) were identified with high combined scores. These processes suggest that the regulation and promotion of miRNA transcription and metabolic activities may play crucial roles in the progression of inflammatory diseases, particularly psoriasis. In the Molecular Function category, GO terms such as RNA polymerase II-specific DNA-binding transcription factor binding (GO:0061629), DNA-binding transcription factor binding (GO:0140297), and kinase binding (GO:0019900) were found to be significant. These molecular functions are involved in gene expression regulation and cell signaling, both of which are critical in inflammatory responses and abnormal skin cell proliferation. For the Cellular Component category, terms including intracellular membrane-bounded organelle (GO:0043231), nucleus (GO:0005634), and cyclin-dependent protein kinase holoenzyme complex (GO:0000307) were identified. These components are closely associated with abnormal skin cell proliferation and inflammatory responses. In particular, dysfunctions in the CDK holoenzyme complex, which is involved in cell cycle regulation, may contribute to the excessive skin cell proliferation observed in psoriasis.

### 3.4. Analysis of Key Active Compounds in Selected Herbal Candidates

We conducted an analysis of the active compounds and core mechanisms of the four herbal candidates—*Piperis longi fructus*, *Pini koraiensis semen*, *Schisandrae fructus*, and *Cnidi fructus*—that were identified as potential novel treatments for psoriasis. To assess the potential impact of each herb’s active compounds on psoriasis, correlation scores were calculated between the diffusion profiles of each herb’s active compounds and psoriasis-related proteins. Based on these scores, up to three active compounds were selected for further analysis. A multi-layer network was then constructed by combining protein target data for both the selected compounds and psoriasis-related proteins, alongside data on their biological functions. The most influential proteins and biological functions in both the herbal compounds and psoriasis-related mechanisms were extracted from the network and visualized. This network reflected key pathways and mechanisms involved in psoriasis.

**Figure 4:**
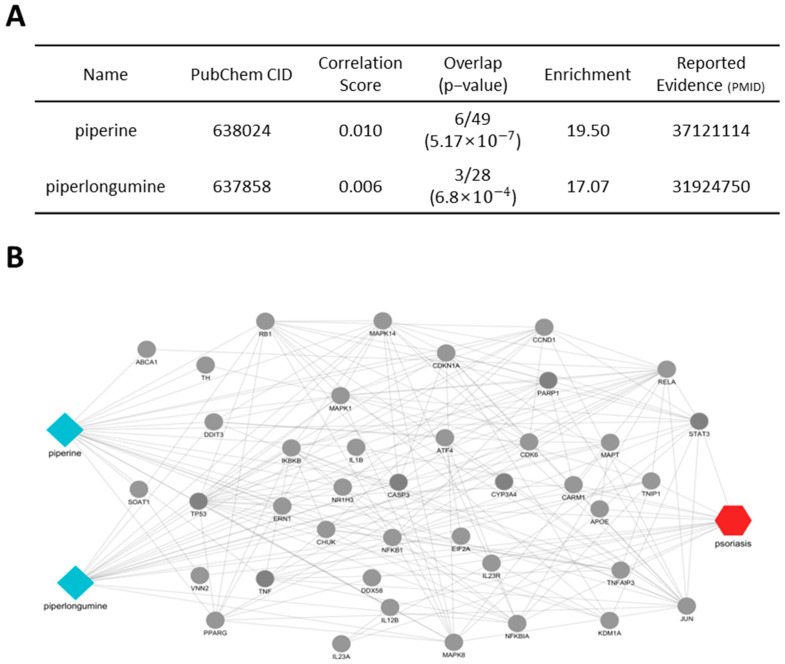
Potential active compounds and key targets of *Piperis longi fructus* in psoriasis. (**A**) The network shows two active compounds, piperine and piperlongumine (blue diamonds), interacting with key psoriasis-related proteins (grey circles). The psoriasis node is represented by the red hexagon, connected to relevant proteins and pathways. (**B**) The accompanying table provides details about these compounds with their reported evidence.

First, the active compounds in *Piperis longi fructus* were analyzed, and the relationships between these compounds and their protein targets were visualized (Figure 4A). The active compounds piperine and piperlongumine were found to be connected to several psoriasis-related protein targets, including STAT3, NFKBIA, TNF, TP53, IL12B, and IL1B (Figure 4B). These proteins have been recognized for their critical roles in psoriasis. STAT3 was shown to regulate inflammatory cytokine signaling and Th17 cell differentiation, while TNF and IL1B were found to contribute to the induction of inflammatory cytokines and amplification of immune responses. NFKBIA was identified as a regulator of inflammation by inhibiting NF-κB activity, and TP53 was associated with the regulation of cell survival and damage responses. Additionally, IL12B was linked to the modulation of Th1 immune responses, which are known to play a significant role in psoriasis-related inflammation. These results indicate that *Piperis longi fructus* could be involved in modulating inflammatory pathways associated with psoriasis.

**Figure 5:**
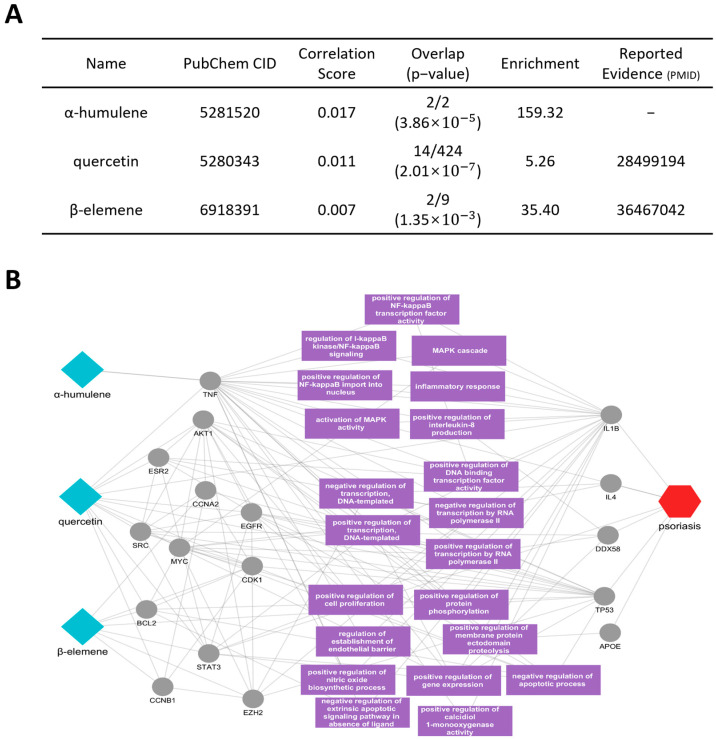
Potential active compounds and key targets of *Pini koraiensis semen* in psoriasis. (**A**) The network shows three active compounds, α-humulene, quercetin, and β-elemene (blue diamonds), interacting with key psoriasis-related proteins (grey circles). The psoriasis node is represented by the red hexagon, connected to relevant proteins and pathways. (**B**) The accompanying table provides details about these compounds with their reported evidence.

Next, we conducted an analysis of the active compounds and core mechanisms of *Pini koraiensis semen*. For this analysis, we selected the three compounds from *Pini koraiensis semen*—α-humulene, quercetin, and β-elemene—by prioritizing their correlation scores, while also considering their *p*-values (Figure 5A). The key elements impacting the network between these compounds and psoriasis were extracted, and their relationships were visualized (Figure 5B).

The analysis revealed that α-humulene was directly linked to the psoriasis-related proteins IL1B and TNF, while quercetin was associated with IL1B, DDX58, and TP53. Additionally, β-elemene was found to interact with proteins such as EZH2, CCNB1, CCNA2, BCL2, STAT3, and CDK1. These protein targets were shown to be involved in diverse interaction networks associated with psoriasis-related proteins. Further investigation indicated that these targets are related to several biological functions, including inflammation and signal transduction. Specifically, these proteins were involved in critical pathways, including NF-κB signaling, MAPK activation, and inflammatory responses. This includes the positive regulation of NF-κB transcriptional activity, its movement into the nucleus and interleukin-8 production. These results indicate that *Pini koraiensis semen* can modulate key biological pathways related to inflammation and immune responses.

**Figure 6:**
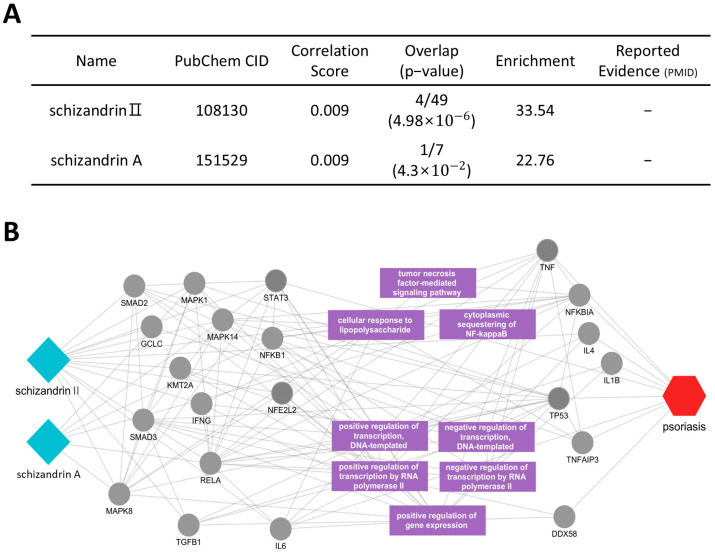
Potential active compounds and key targets of *Schisandrae fructus* in psoriasis. (**A**) The network shows two active compounds, schizandrin II and schizandrin A (blue diamonds), interacting with key psoriasis-related proteins (grey circles). The psoriasis node is represented by the red hexagon, connected to relevant proteins and pathways. (**B**) The accompanying table provides details about these compounds with their reported evidence.

Similarly, we analyzed the active compounds and mechanisms of *Schisandrae fructus*, with a focus on schizandrin II and schizandrin A (Figure 6A). The core mechanisms of schizandrin II and schizandrin A were analyzed, and key elements within the multi-layer network with a significant impact on psoriasis were extracted. The relationships between these elements were visualized (Figure 6B). The analysis demonstrated that schizandrin II directly influenced psoriasis-related proteins such as IL-4, IL-6, STAT3, and NFKBIA, while schizandrin A modulated the NFKBIA protein. These proteins contribute to essential biological functions associated with the pathological features of psoriasis, including the tumor necrosis factor-mediated signaling pathway, cytoplasmic sequestering of NF-κB, and the cellular response to lipopolysaccharide. These findings indicate that schizandrin II and schizandrin A possess the potential to regulate the activity of proteins involved in the pathophysiology of psoriasis.

**Figure 7:**
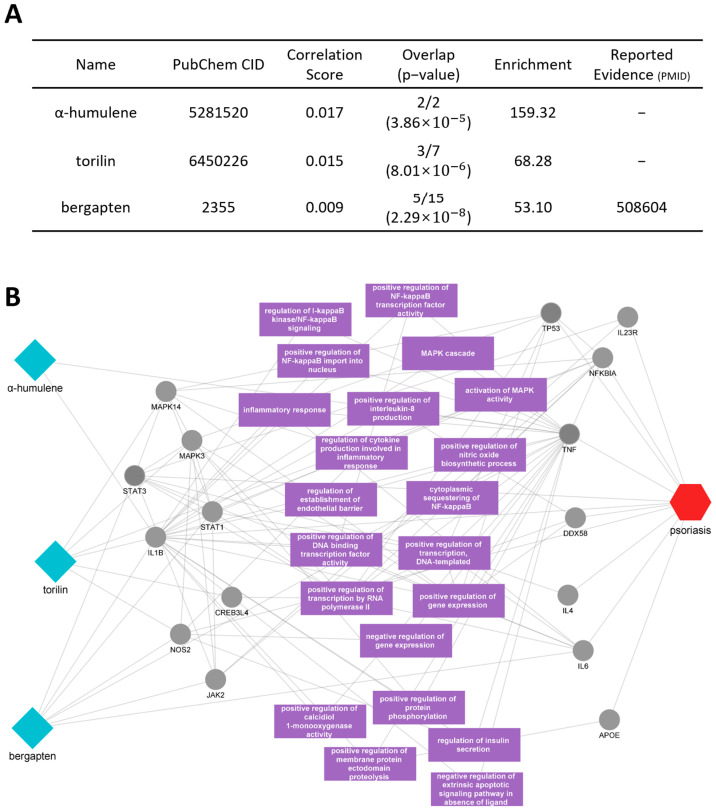
Potential active compounds and key targets of *Cnidi fructus* in psoriasis. (**A**) The network shows three active compounds, α-humulene, torilin, and bergapten (blue diamonds), interacting with key psoriasis-related proteins (grey circles). The psoriasis node is represented by the red hexagon, connected to relevant proteins and pathways. (**B**) The accompanying table provides details about these compounds with their reported evidence.

We conducted an additional analysis of the active compounds and key mechanisms of *Cnidi fructus* (Figure 7A). Based on the compound analysis results, we focused on the three top-ranking compounds—α-humulene, torilin, and bergapten—which exhibited high correlation scores and significant protein overlap, to evaluate their potential contributions to psoriasis treatment (Figure 7B). The target proteins of these active compounds, including IL1B, TNF, STAT3, IL6, NOS2, and NFKBIA, were found to be directly associated with psoriasis-related disease proteins. Additionally, proteins such as MAPK3, MAPK14, JAK2, and STAT1 were identified as having a direct impact on psoriasis-related disease proteins. These target proteins were shown to regulate several biological processes, particularly those associated with inflammation, including the activation of the NF-κB signaling pathway, the MAPK cascade, and the positive regulation of nitric oxide biosynthesis. The identification of these interactions highlights the potential role of *Cnidi fructus* in modulating key inflammatory pathways relevant to psoriasis pathogenesis.

## 4. Discussions

In this study, we systematically analyzed the complex interactions between herbs, compounds, and target proteins using a multiscale network-based framework. Through this approach, we identified potential herbal candidates and active compounds with therapeutic potential for psoriasis. We discovered *Piperis longi fructus, Pini koraiensis semen, Schisandrae fructus*, and *Cnidi fructus* as novel candidates for psoriasis treatment, which had not been previously reported in the literature. These herbs showed high correlation scores within the psoriasis-related protein network, suggesting their potential as promising therapeutic agents. Additionally, we identified well-known herbs such as *Illici veri fructus*, *Magnoliae cortex*, and *Scutellariae radix*, which have been previously studied for their anti-psoriatic effects, thereby reinforcing the validity of the network pharmacology approach. Our findings provide a deeper understanding of the effects of herbs on psoriasis and propose new herbal candidates for therapeutic development.

The results of the candidate herbs identified in this research aligned with findings from previous studies on psoriasis, thereby demonstrating the effectiveness of the network-based approach. Specifically, we found that *Illici veri fructus*, *Magnoliae cortex*, and *Scutellariae radix* exhibited high correlation scores, confirming them as promising therapeutic candidates. These herbs have already been reported to be effective in treating psoriasis in prior research. This outcome can be attributed to the diffusion profile analysis measured through a random walk algorithm within the multiscale network, which comprehensively considered not only protein–protein interactions but also the relationships between proteins and biological functions. These findings highlight the utility of network-based approaches in elucidating the complex mechanisms by which herbs and their active compounds influence diseases through multiple pathways. Furthermore, they make a significant contribution to proposing novel therapeutic strategies for complex diseases such as psoriasis.

We also identified *Piperis longi fructus*, *Pini koraiensis semen*, *Schisandrae fructus*, and *Cnidi fructus* as new herbal candidates. Network pharmacological analysis suggested their potential as novel therapeutic options for future clinical applications. The active compounds of *Piperis longi fructus*, *Pini koraiensis semen*, and *Cnidi fructus* have been previously studied for their potential in treating psoriasis. Piperine inhibited the phosphorylation of STAT3, thereby reducing psoriatic skin inflammation [28]. Piperlongumine and quercetin modulated NF-κB signaling, exerting strong anti-inflammatory effects [29,30]. β-elemene induced apoptosis in psoriatic keratinocytes and suppressed inflammation by downregulating pro-inflammatory cytokine expression in M1 macrophages [31]. Bergapten (5-MOP) acted as a photosensitizer in psoriasis treatment, effectively clearing psoriatic lesions [32]. Collectively, these findings not only validate the accuracy of our network-based predictive model but also highlight the potential of less-studied herbs as innovative therapeutic candidates for the treatment of psoriasis.

Additionally, we identified schizandrin II, schizandrin A, α-humulene, and torilin from *Schisandrae fructus*, *Pini koraiensis semen*, and *Cnidi fructus* as novel active compounds. α-humulene from *Pini koraiensis semen* was found to interact with psoriasis-related proteins such as IL1B and TNF, which play crucial roles in inducing inflammatory cytokines and amplifying immune responses. This suggests that α-humulene may contribute to the inflammatory mechanisms of psoriasis. Schizandrin II and Schizandrin A from *Schisandrae fructus* were shown to interact with target proteins, including IL-4, IL-6, STAT3, and NFKBIA, which are involved in psoriasis-related pathways such as tumor necrosis factor-mediated signaling, cytoplasmic sequestration of NF-κB, and cellular response to lipopolysaccharide. These findings indicate that the components of *Schisandrae fructus* have the potential to modulate the function of psoriasis-related proteins. Furthermore, α-humulene and torilin from *Cnidi fructus* were identified as targeting psoriasis-related proteins, including IL1B, TNF, NOS2, and NFKBIA. These proteins are closely linked to inflammatory regulatory pathways such as NF-κB signaling, the MAPK cascade, and positive regulation of nitric oxide biosynthesis, suggesting that the components of *Cnidi fructus* may have a significant role in psoriasis treatment.

We identified the major targets of active compounds that may influence psoriasis through a key mechanism analysis on the multiscale network. We confirmed that STAT3 was the primary target in this mechanism analysis. Previous reports showed that STAT3 hyperactivation regulates inflammatory pathways that play a crucial role in the onset of psoriasis, and inhibiting STAT3 can effectively alleviate psoriasis symptoms by reducing Th17 cell differentiation and inflammatory cytokine signaling [33]. We also found that TNF was a major target. TNF is a key cytokine that mediates inflammatory responses and immune cell activation, and it can induce other cytokines such as IL-24. By inhibiting TNF, it is possible to block inflammatory pathways and effectively relieve psoriasis symptoms [34]. NFKBIA was identified as a potential target due to its ability to inhibit NF-κB activity, which regulates NF-κB-mediated inflammatory responses and cell proliferation, making it effective in psoriasis treatment [35]. IL-6 was found to activate the STAT3 and Th17 pathways in psoriasis, promoting inflammation and keratinocyte proliferation. Thus, targeting IL-6 could be effective in treating psoriasis [36]. We also identified that key biological functions, such as NF-κB and the MAPK cascade, were significantly involved in the therapeutic mechanisms of active compounds. NF-κB in particular acts as a crucial transcription factor regulating inflammatory responses, cell proliferation, and cell survival in psoriasis. Excessive activation of NF-κB amplifies these processes abnormally, directly contributing to psoriatic lesions. Additionally, the MAPK pathway plays a pivotal role in the pathological progression of psoriasis by regulating keratinocyte proliferation and differentiation, the production of inflammatory cytokines and chemokines, and the recruitment and activation of immune cells, including Th1 and Th17 cells, through the activation of downstream pathways such as p38, ERK, and JNK in psoriatic lesions [37].

This study has several limitations that need to be addressed in future research. First, the data used for constructing the network, including protein targets and their interactions, may be incomplete or subject to inaccuracies. Integrating more comprehensive and up-to-date datasets in the future could enhance the reliability and comprehensiveness of the network. Second, although the methodologies applied in this study are promising, they may require further refinement and validation. Utilizing alternative network analysis techniques or machine learning approaches could potentially improve the predictive accuracy of active compounds and their therapeutic effects. Despite these limitations, our constructed network effectively reflected known therapeutic effects of certain herbs, demonstrating the robustness and novelty of our approach.

## Figures and Tables

**Table 1:** Identified herbal candidates for psoriasis treatment based on a network analysis.

Name	Correlation Score	Overlap (*p*-Value)	Enrichment	Reported Evidence (PMID)
*Piperis longi fructus*	0.0134	5/50 (6.76 × 10^−5^)	11.38	-
*Illici veri fructus*	0.0082	2/11 (3.90 × 10^−3^)	20.69	23872327
*Pini koraiensis semen*	0.0078	6/50 (4.04 × 10^−6^)	13.65	-
*Magnoliae cortex*	0.0077	4/50 (9.00 × 10^−4^)	9.104	38541664
*Schisandrae fructus*	0.0076	4/25 (5.85 × 10^−5^)	18.20	-
*Cnidi fructus*	0.0075	6/50 (4.04 × 10^−6^)	13.65	-
*Inulae heleni radix*	0.0069	3/50 (9.30 × 10^−3^)	6.82	-
*Anemarrhenae rhizoma*	0.0069	7/48 (1.48 × 10^−7^)	16.59	-
*Liriopis seu ophiopogonis tuber*	0.0066	8/50 (8.22 × 10^−9^)	18.20	-
*Scutellariae radix*	0.0065	8/50 (8.22 × 10^−9^)	18.20	36271574

**Table 2:** KEGG pathway enrichment analysis of shared target proteins from psoriasis-related herbs.

Term	Overlap (*p*-Value)	Odds Ratio	Combined Score	Target Proteins
Osteoclast differentiation	26/127 (1.22 × 10^−24^)	23.20	1277.9	CSF1;NCF4;TNFRSF11B;TNF;RELA;IKBKB;MAPK8;AKT1;MAPK1;JAK1;JUN;TGFB1;CHUK;IFNB1;CYBA;MITF;FOS;MAPK14;NFKB1;NFKBIA;IL1A;CREB1;IFNG;IL1B;TRAF6;PPARG
NF-κB signaling pathway	21/104 (5.69 × 10^−20^)	22.32	989.1	CD40;CHUK;PARP1;PRKCB;XIAP;PTGS2;TNF;NFKB1;RELA;ICAM1;NFKBIA;IKBKB;CD40LG;IRAK1;PLAU;IL1B;TRAF6;BCL2;PLCG1;TLR4;MYD88
Cell cycle	20/124 (4.75 × 10^−17^)	16.87	634.1	RB1;SMAD2;GSK3B;SMAD4;CDKN1A;SMAD3;TGFB1;CDKN1B;CCND3;CCNB1;CCND2;CDK6;CCND1;CDK4;CDC27;CDK2;CDK1;E2F1;TP53;ATR
PPAR signaling pathway	11/74 (1.51 × 10^−9^)	14.75	299.7	FABP1;CPT1A;FABP3;FABP4;FABP5;MMP1;LPL;PPARG;ACADM;PPARA;PPARD
NOD-like receptor signaling pathway	24/181 (3.49 × 10^−18^)	13.61	547.4	JUN;CHUK;IFNB1;XIAP;CYBA;MAPK14;TNF;NFKB1;RELA;NFKBIA;IKBKB;IL6;MAPK8;CASP8;IL1B;TRAF6;BCL2;CASP1;NLRP3;MAPK1;PKN1;TLR4;MYD88;JAK1
Arachidonic acid metabolism	6/61 (9.82 × 10^−5^)	9.03	83.3	CYP2C9;ALOX5;CYP2E1;CYP2C19;PTGS2;PTGS1
TNF signaling pathway	28/112 (4.30 × 10^−29^)	30.35	1982.8	ATF2;CSF1;PTGS2;TNF;RELA;ICAM1;IKBKB;CASP7;MAPK8;CASP8;CASP3;AKT1;MAPK1;JUN;CHUK;IFNB1;MMP3;FOS;MAPK14;MMP9;NFKB1;NFKBIA;CXCL10;IL6;CREB1;IL1B;FAS;ATF4
Chemokine signaling pathway	18/192 (2.51 × 10^−11^)	8.96	218.7	GSK3B;CHUK;PRKCB;SRC;BAD;STAT3;NFKB1;PTK2;RELA;NFKBIA;IKBKB;CXCL10;PAK1;CXCR3;AKT1;MAPK1;PLCG1;JAK2
Cytokine–Cytokine receptor interaction	22/295 (1.40 × 10^−11^)	7.07	176.7	IL10;CD40;TGFB1;CSF3R;CSF1;IFNB1;EPO;TNFRSF11B;TNF;IL2;IL4;IL1A;CXCL10;CD4;IL6;BMP2;CD40LG;IFNG;IL1B;CXCR3;IL12B;FAS
Oocyte meiosis	7/129 (1.00 × 10^−3^)	4.75	32.6	AR;CCNB1;CDC27;CDK2;CDK1;MAPK1;MAPK14

## Data Availability

The datasets supporting the conclusions of this article are included within the article.
